# Yeast model identifies *ENTPD6* as a potential non-obstructive azoospermia pathogenic gene

**DOI:** 10.1038/srep11762

**Published:** 2015-07-08

**Authors:** Qian Wang, Chao Liu, Chaoming Tang, Huiping Guo, Yujiao Liu, Lina Wang, Haichao Zhao, Yongliang Shang, Yang Wen, Yuan Lin, Tao Zhou, Zuomin Zhou, Wen Dong, Zhibin Hu, Xuejiang Guo, Jiahao Sha, Wei Li

**Affiliations:** 1 State Key Laboratory of Reproductive Biology, Institute of Zoology, Chinese Academy of Sciences, Beijing 100101, China; 2 State Key Laboratory of Reproductive Medicine, Collaborative Innovation Center of Genetics and Development, Nanjing Medical University, Nanjing 210029, China; 3 Department of Histology and Embryology, Nanjing Medical University, Nanjing 210029, China; 4 University of Chinese Academy of Sciences, Beijing 100049, China; 5 College of Marine Life, Ocean University of China, Qingdao 266003, China

## Abstract

**Approximately ten percent of male infertility is caused by non-obstructive azoospermia (NOA), but the etiologies of many NOA remain elusive. Recently, a genome-wide association study (GWAS) of NOA in Han Chinese men was conducted, and only a few genetic variants associated with NOA were found, which might have resulted from genetic heterogeneity. However, those variants that lack genome-wide significance might still be essential for fertility. Functional analysis of genes surrounding these variants in** ***Drosophila*** **identified some spermatogenesis-essential genes. As a complementary method of** ***Drosophila*** **screening, SK1 background** ***Saccharomvces cerevisiae*** **was used as a model to screen meiosis-related genes from the NOA GWAS data in this study. After functional screening,** ***GDA1*** **(orthologous to human*****ENTPD6*****) was found to be a novel meiosis-related gene. The deletion of** ***GDA1*** **resulted in the failure of yeast sporulation. Further investigations showed that Gda1p was important for pre-meiotic S phase entry. Interestingly, the meiotic role of Gda1p was dependent on its guanosine diphosphatase activity, but not it’s cytoplasmic, transmembrane or stem domains. These yeast data suggest that** ***ENTPD6*** **may be a novel meiosis-associated NOA-related gene, and the yeast model provides a good approach to analyze GWAS results of NOA.**

Infertility, which is a severe threat for the continuation of humans, affects one-sixth of couples worldwide^1^,^2^. Approximately half of the infertility cases are attributed to the male^2^,^3^. In male infertility, 10–15% of cases are classified as azoospermia, and 60% of these cases are non-obstructive azoospermia (NOA), which generally affects 1% of the male population^4^,^5^,^6^. Recently, some genetic factors, such as single nucleotide polymorphisms (SNPs) and other common structural variants, have been reported to be associated with NOA^7^,^8^,^9^; However, the etiology of most of the NOA remains largely unknown.

The genome-wide association study (GWAS) represents a powerful tool for investigating the genetic architecture of complex human diseases and provides a good approach to study the associations between SNPs and traits such as human diseases^10^,^11^. Although GWASs have successfully identified loci that influence a wide variety of human diseases, many of these results are either inconsistent or have failed to be independently validated^12^,^13^, possibly due to high genetic heterogeneity. These loci may still indicate genes important for the disease. Systematically functional analyses are expected to help identify genes that are involved in human diseases. Limited by material and financial resources, it is difficult to perform large-scale functional genomic analysis of the pathogenic genes identified by GWASs in mammals. Therefore, an efficient and convenient method is needed to functionally verify the associations of GWAS results with specific diseases in the post-GWAS era.

As a simple single-cell eukaryote, *Saccharomvces cerevisiae* is widely used as a model organism in biological research and has offered valuable knowledge of the genetics and basic cellular processes that are evolutionarily conserved with higher eukaryotes, such as the cell cycle, DNA replication, recombination, metabolism, aging and meiosis^14^,^15^,^16^,^17^. Many of these results have been directly extended to mammalian systems, thus providing an important tool in understanding complex human diseases^18^,^19^,^20^. Because of its powerful capacity for genetic manipulation and relative low cost in culturing, yeast has been developed as a very important system for annotating gene function, functional genomics and drug discovery, and it is suitable for uncovering the basic functions of the genes implicated in some human diseases^20^,^21^,^22^,^23^.

To detect the SNPs associated with NOA, we performed a large-scale genome-wide association study in Han Chinese men, and 103 SNPs were found to be associated with NOA, with *p* <10^−5^ 24,25,26 in the GWAS scan, but failed in replications. Functional screening of the genes in *Drosophila* orthologous to those around these SNPs by *in vivo* RNA interference (RNAi) identified approximately 32% of the analyzed *Drosophila* genes to be essential for male fertility^26^. However, because of the lack of chromosome recombination in *Drosophila* spermatogenesis^27^, our previous work might have missed some meiosis-related NOA-associated genes. As a classical model for meiotic studies^17^, functional genomic screening in *Saccharomvces cerevisiae* provides an efficient and convenient method to identify meiosis-associated genes that might be evolutionarily conserved from yeast to humans. We identified 9 yeast homologs as potential human NOA pathogenic genes by bioinformatics analysis, one of which, *MSH5*, has been reported to be important for meiosis^28^,^29^. After deleting those non-essential genes in SK1 background yeast strains, we found that one gene was required for yeast sporulation. Similar to human NOA, the deletion of *GDA1* inhibited gametogenesis. In the *GDA1* deletion strain, premeiotic DNA replication was blocked and Sic1p was stabilized, which suggested that Gda1p is primarily required for G1 to pre-meiotic S phase transition. The function of Gda1p in entering the pre-meiotic S phase is dependent on its guanosine diphosphatase activity, but not its glycosylation modification, cytoplasmic, transmembrane or stem domains. Therefore, *ENTPD6*, the human ortholog of *GDA1*, may be a NOA pathogenic gene.

## Results

### Identification of potential non-obstructive azoospermia pathogenic genes by functional screening in yeast

**Figure 1:**
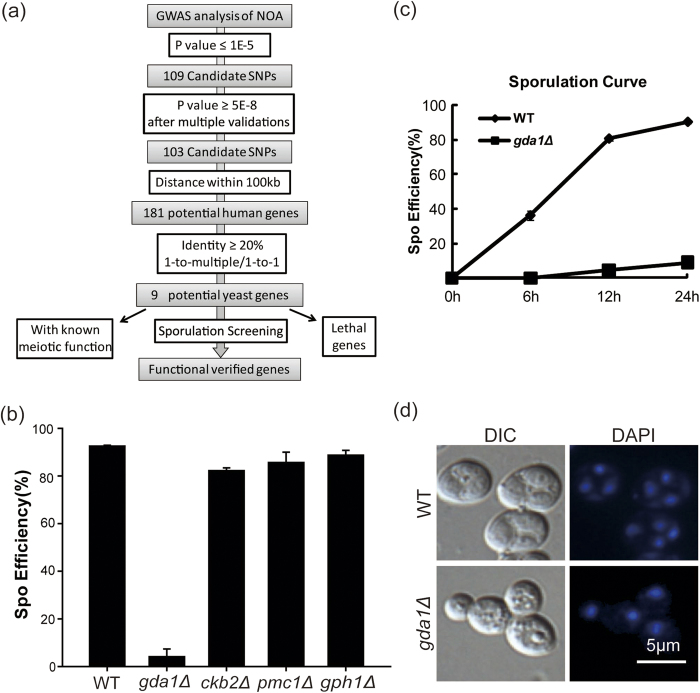
Identification of potential non-obstructive azoospermia pathogenic genes by functional genomic screening in yeast. (**a**) Flow chart of the screening strategy. The selection criteria for candidate genes in *Saccharomvces cerevisiae* included a tSNP with an association P-value < 10^−5^ and a P-value ≥ 5*10^−8^ after multiple validations, and human genes flanking the tSNPs within 100 kb, homology type (one to many or one to one) and orthology identity >20% were considered. After eliminating the well-studied genes in meiosis and lethal genes, the candidate genes were screened for their sporulation efficiency after deletion. (**b**) The sporulation efficiency of the yeast in which candidate genes were deleted. Wild type and candidate gene deletion stains were incubated in sporulation medium for 24 hrs. Sporulation efficiency was the percentage of cells induced to sporulate that became dyads and tetrads by staining with DAPI. (**c**) The *gda1Δ* strain showed a decrease in sporulation efficiency compared with the WT strain. A sporulation time course indicated the percentage of cells/asci with dyads and tetrads in the *gda1Δ* and WT strains. Diploid yeast cells were deprived of nutrients, induced to enter sporulation synchronously, and stained with DAPI at different times post-induction. (**d**) WT and *gda1Δ* spores were stained with DAPI to show the decrease of sporulation efficiency.

A recent study showed that approximately 32% of GWAS SNPs are located in deoxyribonuclease I hypersensitive sites (DHSs), which are markers of regulatory DNA that can regulate genes within 100 kb^30^. Thus, we considered genes flanking the tSNPs (Tag Single Nucleotide Polymorphisms) within 100 kb in this study. Our recent work in *Drosophila* has demonstrated that this approach is effective in identifying genes that are essential for male fertility based on SNPs without genome-wide significant associations with human NOA^26^. However, *Drosophila* spermatogenesis does not involve chromosome recombination^27^, which is an important event for human spermatocyte meiosis. Because yeast is the most powerful model to study meiosis, we used yeast in the present study to screen meiosis-related genes from the NOA GWAS data and used the strategy described in Fig. 1a. In summary, 9 candidate orthologous yeast genes were obtained, corresponding to 11 human genes and 7 susceptible tSNPs (Table S1). Among these, *MPP10*, *RFC5*, *RPC19* and *SLD5* were found to be essential to yeast survival, thus prohibiting our further screening by their deletion. *MSH5* was found to be involved in meiosis in yeast^28^,^29^. Finally, 4 genes, *CKB2*, *GDA1, GPH1,* and *PMC1,* were selected and underwent functional analysis in the SK1 background yeast strain, which sporulates faster and more synchronously than other strains and is commonly used for the study of sporulation or meiosis^31^. After deleting these genes by homologous recombination^32^, wild type (WT) and candidate gene deletion stains were deprived of nitrogen and incubated in sporulation medium for 24 hrs, and the sporulation efficiency was detected by staining with 4’,6-diamidino-2-phenylindole (DAPI). We found that the sporulation efficiency of the *gda1Δ* strain showed a significant decrease compared with that of the WT strain (Fig. 1b–d), which is similar to some NOAs of humans. *ENTPD6* is the orthologous human gene of *GDA1*, and it is a member of the ENTPD family and localizes in the Golgi apparatus^33^. Thus, the functional genomic screening of NOA GWAS data in yeast resulted in the identification of *ENTPD6* as a potential pathogenic gene of NOA.

### *GDA1* is required for pre-meiotic S phase entry

**Figure 2:**
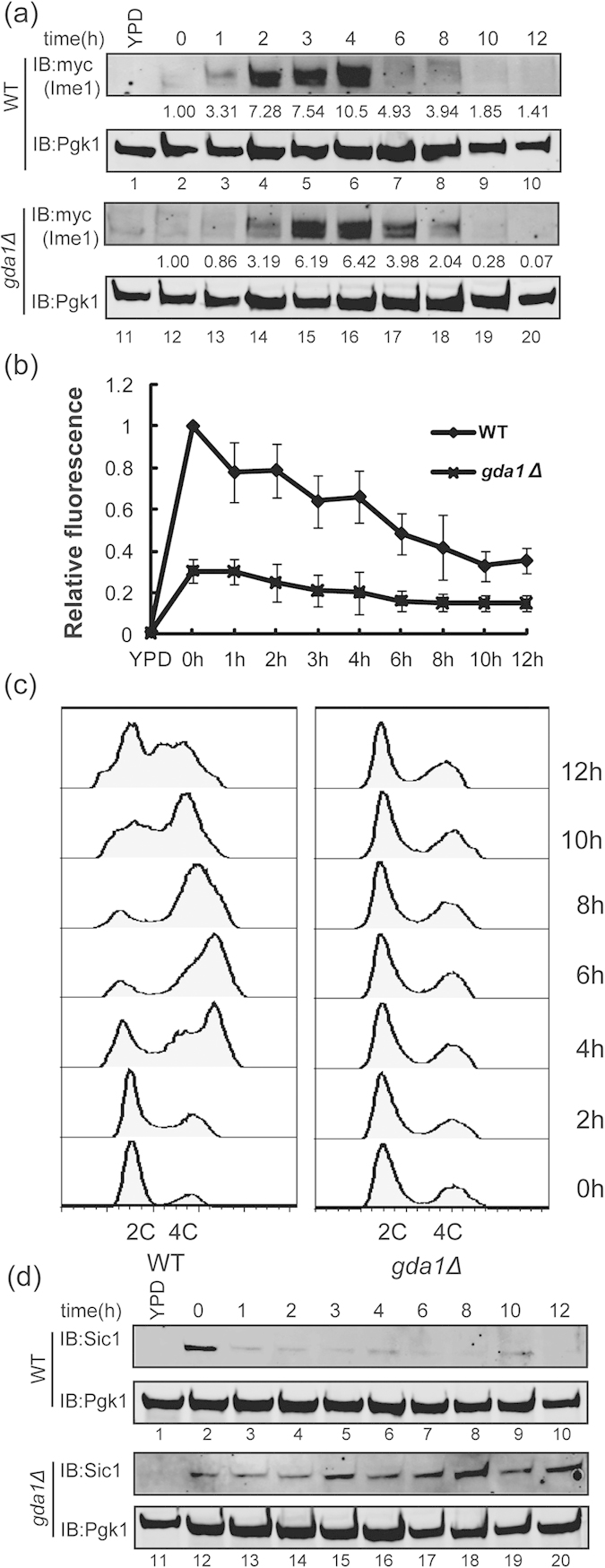
*GDA1* is required for pre-meiotic S phase entry. The expression of Ime1p was only slightly delayed in the *gda1Δ* strain during sporulation. WT or *gda1Δ* strains expressing the *IME1*-3×myc allele were incubated in sporulation medium and samples were collected at different times. The expression of Ime1p-3×myc over time was analyzed by immunoblotting with anti-Myc antibody. Pgk1p served as a loading control. Full-length blots/gels are presented in Supplementary Figure 5. (**b**) Real-time PCR analysis of the *IME1* expression level in WT and *GDA1* deletion strains. (**c**) The pre-meiotic DNA replication was inhibited in the *gda1Δ* strain during sporulation. WT or *gda1*Δ strains were incubated in sporulation medium and samples were collected at different times after induction. DNA content was analyzed by flow cytometry to detect pre-meiotic DNA replication (2C to 4C transition). (**d**) The stabilization of Sic1p in the *gda1Δ* strain during sporulation. WT or *gda1*Δ strains were incubated in sporulation medium and samples were collected at different times after induction. The expression of Sic1p over time was analyzed by immunoblotting with specific anti-Sic1 antibody. Pgk1p served as a loading control. Full-length blots/gels are presented in Supplementary Figure 5.

Because the deletion of *GDA1* inhibited sporulation, we next determined which phases of sporulation were affected in the *GDA1* deleted strain. Meiosis in yeast is initiated by the expression of Ime1p, which serves as the master regulatory switch for meiosis^34^,^35^. First, we detected the expression of Ime1p in the *GDA1* deletion strain by generating a 3×Myc tag on the C-terminus of *IME1*. During the sporulation processes, we found that the expression of Ime1p in the *GDA1* deletion strain was only delayed by approximately 1 hr compared with the WT strain (Fig. 2a). Real-time PCR analysis of *IME1* mRNA in WT and the *GDA1* deletion strain showed similar results to the protein level (Fig. 2b). These results suggest that *GDA1* is not the major regulator of meiosis initiation, even though it is involved in this process to some extent. We next detected the pre-meiotic DNA synthesis by flow cytometry analysis to test whether the *GDA1-*null mutant influenced pre-meiotic DNA replication. We found that the pre-meiotic DNA replication was repressed in the *GDA1* deletion strain (Fig. 2c).

The early phase of sporulation begins when cells make the decision to differentiate into spores, they then exit the mitotic cycle in G1 and enter the pre-meiotic S phase^17^. To determine whether the *GDA1-*null mutant influenced the transition from G1 to the pre-meiotic S phase, we detected the expression of Sic1p, which acts as a central G1 to pre-meiotic S phase transition regulator by inhibiting the Clb5,-6/Cdk1 activity^36^,^37^, presents early in meiosis and subsequently disappears when cells enter the pre-meiotic S phase^38^,^39^. Consistent with other reports^38^,^39^, Sic1p was detected at 1–2 hrs after the induction of sporulation and then disappeared in the following stages in the WT yeast strain (Fig. 2d, lanes 2–10). However, in the *GDA1* deletion strain, Sic1p was detected throughout the process of the induction of sporulation (Fig. 2d, lanes 12–20), which indicated that the *GDA1-*null mutant influenced the G1 to pre-meiotic S phase transition. Therefore, we concluded that the *GDA1*-null mutant mainly arrests before the pre-meiotic S phase and *GDA1* is required for pre-meiotic S phase entry.

### The pre-meiotic S phase entry defect could be rescued by the expression of *GDA1* in the *GDA1* deletion strain

**Figure 3:**
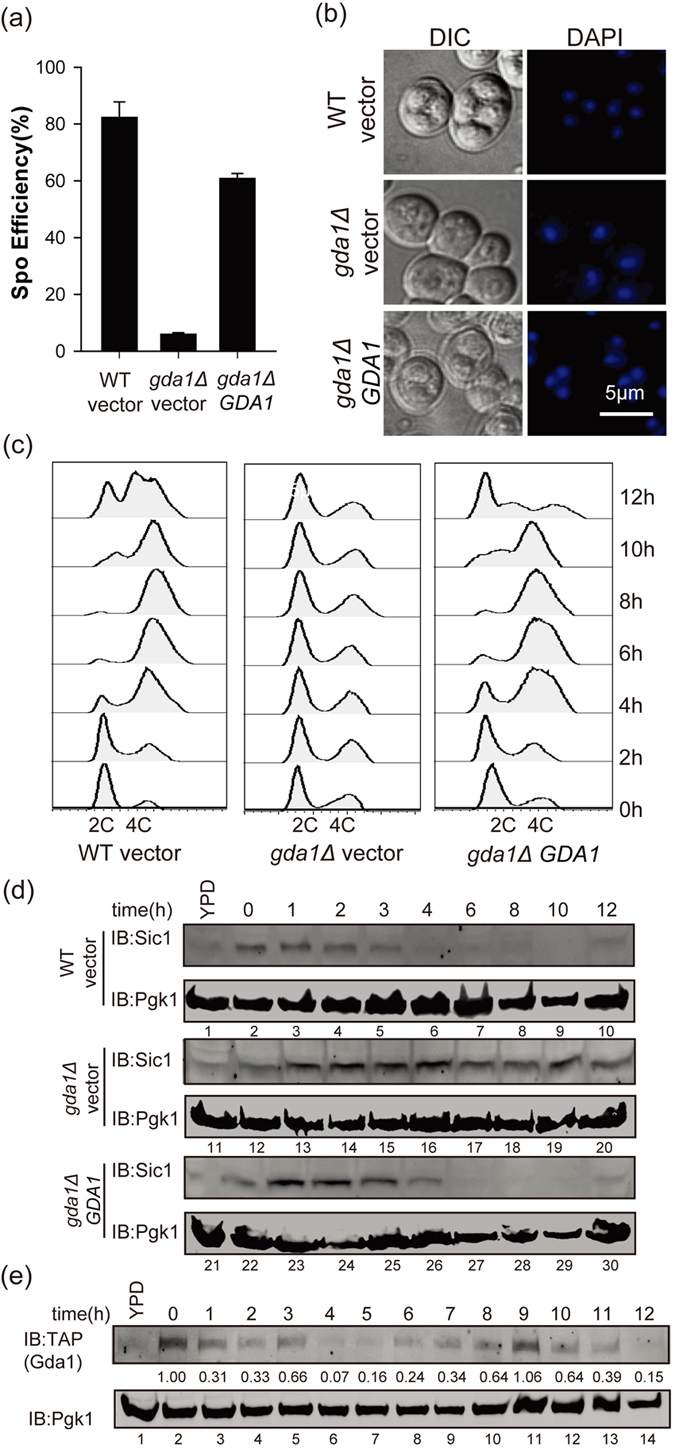
The pre-meiotic S phase entry defect could be rescued by *GDA1* in the *gda1Δ* strain. (**a**) The expression of GDA1 could rescue the sporulation process in the *gda1Δ* strain. The WT strain harbored the empty vector, and the *gda1Δ* strains harbored either the empty vector or *GDA1* under the control of its own promoter. The strains were incubated in sporulation medium for 24 hrs. Sporulation efficiency was assessed by staining with DAPI. (**b**) Microscopic observation of the WT strain harboring the empty vector and the *gda1Δ* strains harboring either the empty vector or *GDA1* under the control of its own promoter after sporulation induction for 24 hrs. (**c**) The expression of *GDA1* could rescue the pre-meiotic DNA replication defect of the *gda1Δ* strain. The WT strain harbored the empty vector, and the *gda1Δ* strains harbored either the empty vector or *GDA1* under the control of its own promoter. The strains were incubated in sporulation medium, and samples were collected at different times after induction. DNA content was analyzed by flow cytometry to detect pre-meiotic DNA replication (2C to 4C transition). (**d**) The expression of of *GDA1* could rescue the stabilization of Sic1p in the *gda1Δ* strain during sporulation. The WT strain harbored empty vector, and the *gda1Δ* strains harbored either empty vector or *GDA1* under the control of its own promoter. The strains were incubated in sporulation medium, and samples were collected at different times after induction. The expression of Sic1p over time was analyzed by immunoblotting with specific anti-Sic1 antibody. Pgk1p served as a loading control. Full-length blots/gels are presented in Supplementary Figure 5. (**e**) The expression of Gda1p during sporulation. The WT strain expressing the *GDA1*-TAP allele was incubated in sporulation medium, and samples were collected at different times after sporulation induction. The expression of Gda1-TAP over time was analyzed by immunoblotting with anti-TAP antibody. Pgk1p served as a loading control. Full-length blots/gels are presented in Supplementary Figure 5.

To further confirm the effect of *GDA1* in entering the pre-meiotic S phase, a *GDA1* expression vector under the control of its own promoter was generated and transformed into a *GDA1* deletion strain. After being incubated in sporulation medium for 24 hrs, we detected the sporulation efficiency of WT vector (containing the empty vector), *gda1Δ* vector (containing the empty vector) and *gda1Δ GDA1* (containing the *GDA1* expression vector under the control of its own promoter) strains, and we found that *GDA1* expression in the *gda1Δ* strain could partially rescue its sporulation defect up to more than 60% compared with the *gda1Δ* vector strain, whose sporulation efficiency remained less than 10% (Fig. 3a). By contrast, the *gda1Δ GDA1* strain could produce spores (Fig. 3b) and complete pre-meiotic DNA replication (Fig. 3c); however, the *gda1Δ* vector strain failed to do so. We then detected the Sic1p expression pattern during sporulation processes in these three strains and found that the Sic1p expression in the *gda1Δ GDA1* strain was similar to that of the WT vector strain (Fig. 3d). These results suggested that the defect of sporulation could be rescued by the expression of *GDA1* in its deletion strain, and *GDA1* indeed played very important roles in entering the pre-meiotic S phase.

We then detected the expression of Gda1p during the process of sporulation by generating a TAP tag on its C-terminus, and we found that Gda1p accumulated in the early phase of sporulation and subsequently decreased in the middle phases at approximately 4–6 hrs (Fig. 3d), which is consistent with its function in the meiotic early phase. Gda1p could also be detected in the late phase of sporulation (Fig. 3e), which may be related to its role in spore wall biogenesis^40^.

### The cytoplasmic, transmembrane and stem domains of Gda1p are not necessary for its role in meiosis

**Figure 4:**
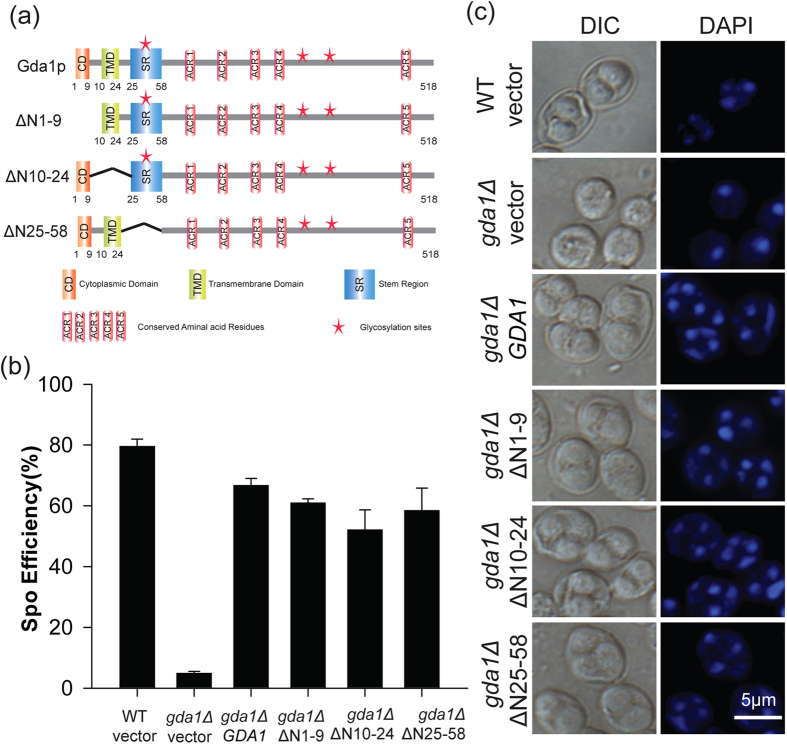
N-terminal domains of Gda1p are not necessary for its role in meiosis. (**a**) Schematic representation of the domains of Gda1p and some mutants, including ΔN1–9 (Δ1–9aa), ΔN10–24 (Δ10–24aa), and ΔN25–58 (Δ25–58aa). CD indicates the cytoplasmic domain; TMD indicates the transmembrane domain; SR indicates the stem region; ACR1–5 indicates the conserved amino acids related to guanosine diphosphatase activity; stars indicate the glycosylation sites. (**b**) The function of *GDA1* in meiosis was independent of its localization-terminal domains. The WT strain harboring the empty vector and the *gda1Δ* strains harboring either the empty vector or *GDA1*, ΔN1–9, ΔN10–24, ΔN25–58 under the control of its own promoter were incubated in sporulation medium for 24 hrs. Sporulation efficiency was determined by staining with DAPI. (**c**) Microscopic observation of the WT strain harboring the empty vector and the *gda1Δ* strains harboring either the empty vector or *GDA1* and its variants under the control of its own promoter after sporulation induction for 24 hrs.

Gda1p is a guanosine diphosphatase that is located in the Golgi, and it is involved in the transport of GDP-mannose into the Golgi lumen by converting guanosine diphosphate (GDP) to guanosine monophosphate (GMP) after mannose is transferred to its substrate. The *GDA1* deletion strain has severe defects in the N- and O-mannosylation of proteins and glycosphingolipids^41^,^42^. Gda1p has a short cytoplasmic domain in its N-terminus. The next domain is a single transmembrane domain, followed by a stem region and a large catalytic luminal domain (Fig. 4a). To further study the functional role of Gda1p in sporulation, we generated three truncations: ΔN1-9 (Gda1 Δ1–9aa, in which the cytoplasmic domain is deleted), ΔN10–24 (Gda1 Δ10–24aa, in which the transmembrane domain is deleted) and ΔN25–58 (Gda1 Δ25–58aa, in which the stem region is deleted) (Fig. 4a). These were transformed into the *gda1Δ* strain. We found that none of the mutants affected the sporulation efficiency compared with the WT *GDA1* (Fig. 4b,c). These results suggested that the function of Gda1p in meiosis is not dependent on these three domains. Additionally, in agreement with our results, it has been reported that the cytoplasmic and transmembrane domains are not necessary for the Golgi localization of Gda1p^43^.

### Glycosylation of Gda1p is not necessary for sporulation

**Figure 5:**
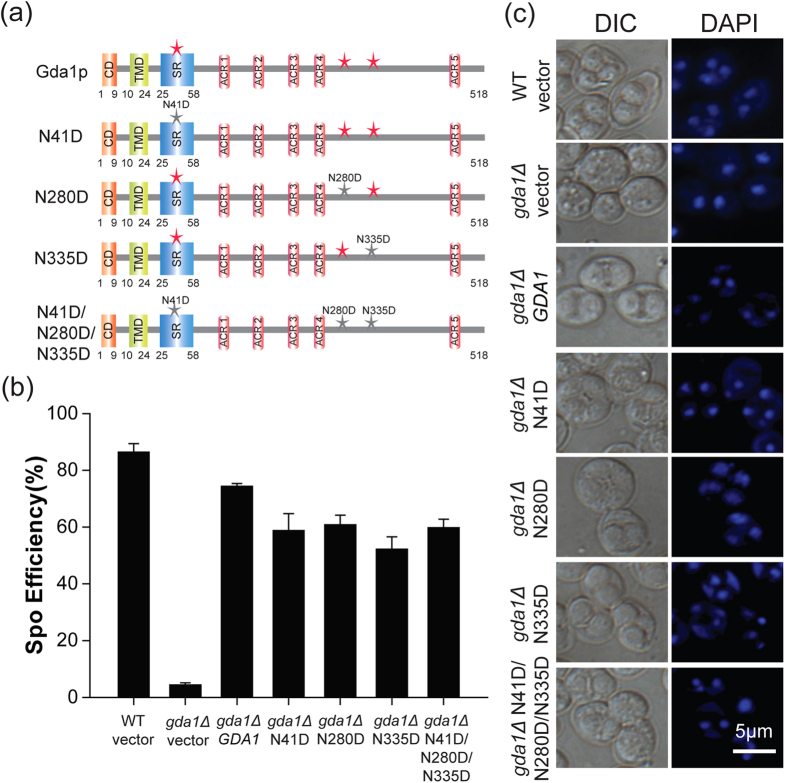
Glycosylation modification of Gda1p is not necessary for yeast sporulation. (**a**) Schematic representation of the mutant glycosylation sites, which included *GDA1* (Gda1p 1–518aa), N41D (Gda1p N41D), N280D (Gda1p N280D), N335D (Gda1p N335D), and N41D/N280D/N335D (Gda1p. N41D/N280D/N335D). (**b**) Glycosylation site mutations of *GDA1* were not necessary for yeast sporulation. The WT strain harboring empty vector and the *gda1Δ* strains harboring the empty vector or *GDA1*, N41D, N280D, N335D, N41D/N280D/N335D under the control of its own promoter were incubated in sporulation medium for 24 hrs. Sporulation efficiency was determined by staining with DAPI. (**c**) Microscopic observation of the WT strain harboring empty vector and the *gda1Δ* strains harboring either the empty vector, WT *GDA1* or glycosylation site mutants under the control of their own promoter after sporulation induction for 24 hrs.

The nucleoside triphosphate diphopshohydrolases (NTPDases) often contain glycosylation sites, and the glycosylation of NTPDase is important for correct protein folding, membrane targeting and activity^44^. As an NTPDase, Gda1p contains three glycosylation sites, which are N41, N280 and N335^45^. To detect whether the glycosylation of Gda1p is involved in its function in sporulation, we abolished its glycosylation by mutating these glycosylation sites to aspartic acids (N41D, N280D and N335D), either separately or together (N41D/N280D/N335D) (Fig. 5a), and transformed these four mutants into the *gda1Δ* strain. By detecting the sporulation efficiency, we found that these mutants did not affect yeast sporulation compared with the control groups (Fig. 5b,c), which indicated that the glycosylation modification of Gda1p is not necessary for its role in meiosis.

### The function of Gda1p in entering the pre-meiotic S phase is dependent on its guanosine diphosphatase activity

**Figure 6:**
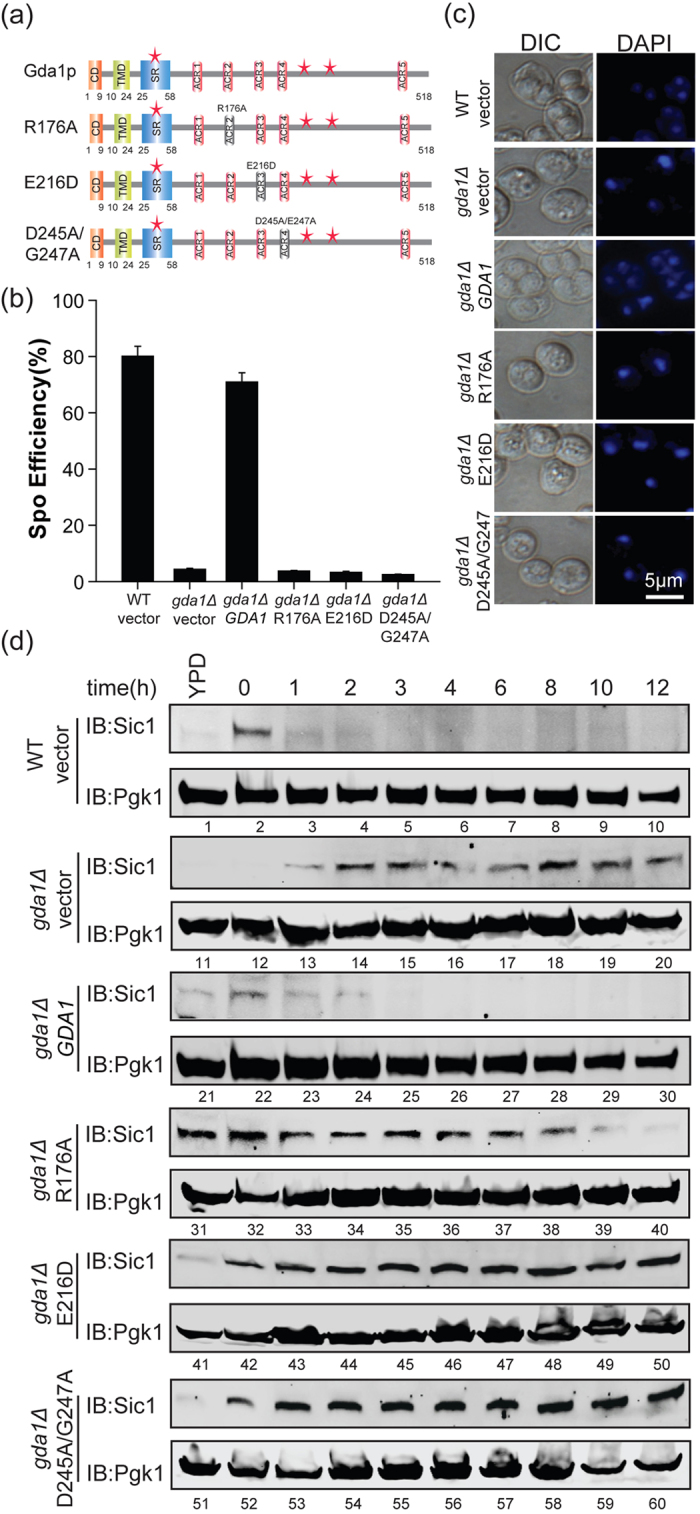
The function of Gda1p in entering the pre-meiotic S phase is dependent on its guanosine diphosphatase activity. (**a**) Schematic representation of the key guanosine diphosphatase activity mutants, which include *GDA1* (Gda1p 1–518aa), R176A (Gda1p R176A), E216D (Gda1p E216D), and D245A/G247A (Gda1p D245A/G247A). (**b**) The disruption of Gda1p guanosine diphosphatase activity induced a decrease in sporulation efficiency compared with the WT strain. The WT strain harboring the empty vector and the *gda1Δ* strains harboring either the empty vector or *GDA1*, R176A, E216D, D245A/G247A under the control of its own promoter were incubated in sporulation medium for 24 hrs. Sporulation efficiency was determined by staining with DAPI. (**c**) Microscopic observation of the WT strain harboring empty vector and the *gda1Δ* strains harboring either the empty vector, WT *GDA1* or key guanosine diphosphatase activity mutants under the control of their own promoter after sporulation induction for 24 hrs. (**d**) The disruption of *GDA1* guanosine diphosphatase activity stabilized Sic1p during sporulation. The WT strain harboring empty vector and the *gda1Δ* strains harboring either empty vector or *GDA1*, R176A, E216D, D245A/G247A mutants under the control of their own promoter were incubated in sporulation medium and samples were collected at different times after induction. The expression of Sic1p over time was analyzed by immunoblotting with specific anti-Sic1 antibodies. Pgk1p served as a loading control. Full-length blots/gels are presented in Supplementary Figure 5.

The NTPDase enzyme activity depends on several strictly conserved motifs, called apyrase conserved regions (ACRs), and some key residues were found to be essential to the activity of these NTPDases^44^,^46^. There are five ACRs in Gda1p, called ACR1–5, and these ACRs are distributed in the lumenal domain of Gda1p^44^. To identify whether the guanosine diphosphatase activity of Gda1p is necessary for its function in sporulation, we selected and mutated R176, E216 and D245/G247 to abolish its enzyme activity, which were named as R176A, E216D and D245A/G247A (Fig. 6a). The expression of these mutants was superior to that of the WT proteins (Fig. S1a), which might have been due to the complementary demand of the activity of this enzyme. After transforming these mutants into the *gda1Δ* strain, we detected the sporulation efficiency and pre-meiotic DNA synthesis. We found that these three mutant strains failed to exhibit pre-meiotic DNA replication and sporulation, similar to the *gda1Δ* strain (Fig. 6b,c, Fig. S1b). We then detected Sic1p expression in these strains, and similar to the *gda1Δ* strain, we found that the Sic1p was stabilized throughout the sporulation process in the enzyme activity-abolished strains (*gda1Δ*R176A, *gda1Δ*E216D and *gda1Δ*D245A/G247A) (Fig. 6d). Therefore, we concluded that the function of Gda1p in entering the pre-meiotic S phase is dependent on its guanosine diphosphatase activity.

## Discussion

To date, hundreds of tag single nucleotide polymorphisms (tSNPs) have been found to be associated with human diseases by genome-wide association studies^10^, and GWAS provides a good approach to study the associations between tSNPs and traits such as major diseases^10^,^11^. However, the results of many GWASs are either inconsistent or have failed to be independently replicated due to the high genetic heterogeneity of the population. Nevertheless, the identified tSNPs may still indicate important genes for the diseases, but most of them still lack systematically functional studies^12^,^13^. The yeast model has been widely used in large-scale functional genomic screenings to reveal the functions of some genes implicated in many human diseases^15^,^18^,^20^,^21^,^22^. Therefore, for NOA, functional genomic screening in yeast may be an efficient and convenient approach to identify genes that are essential for gametogenesis using NOA GWAS results, especially for meiosis, defects in which are important causes of NOA.

Using a strategy to identify meiosis-essential genes in yeast based on SNPs associated with human NOA without genome-wide significance (Fig. 1a), we identified 9 homologs of human genes that were indicated by NOA SNPs in yeast. After eliminating the lethal genes, we found that 1 (25%) of the 4 were required for sporulation, which indicated the usefulness of NOA GWAS data to find meiosis-essential genes. It has been found that ~340 genes (~6% of the yeast genome) are required for sporulation^47^, so our hit rate was higher than that obtained in simple large-scale functional genomic analysis.

In this study, we found that the deletion of *GDA1* resulted in the abolishment of the sporulation process in the SK1 background yeast strain. The *GDA1-*null mutant failed to produce gametes, which is similar to the phenotype of human NOA. Gda1p is a guanosine diphosphatase that is involved in the transport of GDP-mannose into the Golgi lumen by converting GDP to GMP after mannose is transferred to its substrate^41^,^42^. To test whether the products of Gda1p were essential for sporulation, we directly added the product analog of Gda1p, Guanosine 5’-monophosphate disodium salt hydrate (GMP-Na_2_) or Uridine 5’-monophosphosphate disodium salt (UMP-Na_2_), into the YPA medium and sporulation medium. After sporulation, we found that the addition of GMP-Na_2_ and UMP-Na_2_ could not rescue the sporulation defect of the *GDA1* deletion strain (Fig. S2). *KRE2* and its paralog *KTR6* have been reported to be Golgi α1, 2- mannosyltransferases, and they can add the second and third mannose on O-linked glycans and release Gda1p’s substrate GDP from GDP- mannose in the Golgi^48^,^49^,^50^. Therefore, considering the concentration of GDP in the Golgi*, KRE2* and its paralog *KTR6* should be upstream of GDA1. Unexpectedly, both the *KRE2-*null mutant and the *KRE2*/*KTR6* double deletion mutant sporulated as efficiently as the control strain (Fig. S3). All of these results suggested that the nucleoside metabolism-related function of Gda1p may not be essential for sporulation.

It was reported that Gda1p also participates in the N- and O-mannosylation of proteins and glycosphingolipids^41^,^42^. We found that the guanosine diphosphatase activity of Gda1p was essential to its role in the entry into the pre-meiotic S phase by affecting the stability of Sic1p (Fig. 6). Gda1p may affect Sic1p stability in the following ways: 1) Gda1p may directly regulate the glycosylation of Sic1p to promote its degradation, or 2) Gda1p may influence the glycosylation of some other proteins, which are essential for pre-meiotic S phase entry. As far as we know, there is no report on the glycosylation of Sci1p. Thus, we further considered the second indirect possibility; it was reported that the G1-cyclin/Cdk1 complexes catalyzed the phosphorylation of Sic1p during the mitotic G1/S transition, therefore the phosphorylated Sic1p could be recognized and further ubiquitinated by the ubiquitin ligase SCF^Cdc4^ to promote its degradation^51^,^52^. Any molecule related to the above-mentioned process might be targeted by Gda1p to influence the stability of Sicp1. Consistent with this hypothesis, it was reported that Skp1p could be glycosylated to modulate the E3 activity of SCF^Cdc4^ in *Dictyostelium*^53^. Therefore, Gda1p may influence the glycosylation of either Skp1p or any other unknown proteins to promote Sic1p destruction during pre-meiotic S phase entry.

*ENTPD6* is the human ortholog of *GDA1; ENTPD6* is a member of the ENTPD family, which shows UDPase activity and localizes in the Golgi apparatus^33^, and is highly expressed in the testis^54^. It has been reported that protein glycosylation plays a very important role during mammalian reproduction^55^. Therefore, *ENTPD6* may be a NOA pathogenic gene and might be involved in meiosis. The mechanistic study and functional analysis of *GDA1* in yeast have provided important clues for further exploring the role of *ENTPD6* in human spermatogenesis.

In contrast to our results, it was reported that *GDA1* deletion results in the increase of the sporulation efficiency in the S288C background yeast strain^56^. To test their results, we created a *GDA1* deletion strain in the BY4743 background and tested its sporulation efficiency (Fig. S4). We found that the *GDA1-*null mutant showed an increase in sporulation efficiency compared with the WT strain, which was entirely different from the SK1 background strain used in this study. This result suggests that there might have been a redundancy of the function of Gda1p in the S288C background. This result is very similar to the pathology of some human diseases and hints that *ENTPD6* mutations may result in NOA in some people but not in other populations. The detailed mechanism underlying this phenomenon needs to be further investigated in future work.

## Materials and Methods

### Screening of candidate functional yeast gene orthologs based on SNPs associated with human NOA

The tSNPs associated with human NOA (with *P* values less than 10^−5^) were extracted from previous GWAS of NOA performed in Han Chinese men^24^. To obtain candidate fertility-related genes, we considered human genes located within 100 kb upstream or downstream of the SNPs. We then selected the corresponding unique homologous yeast genes (orthologous type: one-to-many or one-to-one) of the candidate human genes for potential targets to be verified. We only considered yeast genes with at least 20% sequence identity with their human orthologs. The genome backgrounds for humans and yeast were GRCh37 and R64-1-1, respectively. SNPs localizations were batch-obtained from the UCSC genome browser (http://genome.ucsc.edu/). Gene descriptions, genome localizations, and orthologous relationships of the human and yeast genes were annotated via BioMart (http://www.biomart.org/).

### Antibodies

The Myc and FLAG antibodies were purchased from Abmart (Shanghai, China), the Sic1 antibody was purchased from Santa Cruz Biotechnology (Santa Cruz, United States), and the TAP antibody was purchased from Thermo Scientific (Waltham, MA USA). The Pgk1 polyclonal antibody was generated in rabbits using the corresponding recombinant proteins as antigens.

### Strains and Plasmids

All plasmids and yeast strains used in this study are described in Tables S2 and S3.

### Growth and sporulation

Cells were grown in YPD medium (1% yeast extract, 2% peptone, 2% glucose), or YPA medium (1% yeast extract, 2% peptone, 2% potassium acetate). The sporulation was performed as previously described^57^. Cells were grown overnight in liquid YPD medium and diluted in liquid YPA medium to an OD600 of 0.3 and cultured for 10 hr at 30 °C. Cells were harvested and resuspended in sporulation medium (2% potassium acetate) to OD600 of 1.9 and sporulated at 30 °C for different lengths of time. In the Gda1p product addition experiments, various concentrations of guanosine 5’-monophosphate disodium salt hydrate (CAS:5550-12-9) or Uridine 5’-monophosphosphate disodium salt (CAS:3387-36-8) were added into the YPA medium and sporulation medium to test their effects on sporulation.

### DAPI staining

Sporulation was assayed by the microscopic examination of cultures that had been incubated in SPM (sporulation medium) for 24 hrs. Approximately two hundred cells per culture were counted and the percentage of cells that had formed Asci was scored. The nuclear DNA was stained by 4’,6-diamidino-2-phenylindole (DAPI) as previously described^58^. The nuclei were visualized and counted using a Nikon Eclipse Ti microscope.

### Yeast whole-cell extract preparation and immunoblotting analysis

The yeast whole-cell extract preparation was performed as previously described^59^. The samples were collected and resuspended in 30 μl distilled water, and an equal volume of 0.2 M NaOH was added. Next, cells were collected after 10 min of incubation at room temperature, and the supernatant was carefully removed. Approximately 30 μl of SDS-sample buffer (100 mM Tris-HCL, pH6.8, 200 mM DTT, 4% SDS, 0.2% BPB, and 20% glycerol) was added to the pellet, and the cells were resuspended and boiled for 10 min and centrifuged briefly. The extract was loaded onto an SDS-PAGE gel and detected by immunoblotting with primary antibodies. The immunoblotting was performed using a fluorescent dye-labeled secondary antibody (Invitrogen), and the blots were scanned using an Odyssey infrared imager.

### Isolation of RNA from yeast

The RNA isolation was performed as previously described^60^. The samples were collected and resuspended in 400 μl AE buffer (50 mM Na acetate pH 5.3, 10 mM EDTA), and 40 μl 10% SDS was added. The suspension was vortexed for 5 min, and 400 μl fresh phenol was added. The mixture was again vortexed for 5 min and incubated at 65 °C for 4 min; the mixture was rapidly chilled on ice for 5 min and then centrifuged for 2 min at 12000 rpm. The upper aqueous phase was transferred to a fresh tube. Then, phenol and chloroform (1:1) was added and extracted for 5 min at room temperature. After being centrifuged for 5 min at 12000 rpm, the upper aqueous phase was again transferred to a fresh tube, and 40 μl 3 M Na acetate and 2.5 volumes ethanol were added to precipitate RNA. After washing with 80% ethanol, the pellet was dried for 5 min, resuspended in 20 μl DEPC-treated water and stored at −80 °C.

### Real-time PCR for *IME1* mRNA

Real-time PCR was carried out with the Roche Light Cycler® 480II System. cDNA was synthesized by the PrimeScript^TM^ RT Reagent Kit (TaKaRa, RR037A), A 10 μL volume of the system with 5 μL of 2×EvaGreen mix (Applied Biological Materials Inc., MasterMix-S), 0.8 μL of each primer (10 nmol/L), 2 μL of sample cDNA, and 2.2 μL of ddH2O was prepared for amplification. Primer sets for IME1 (5’-GACACAACCACCGATCAAGAAG-3’ and 5’-GATGAGTGGAACGTAGATGCG-3’) and ACT1 (5’-CCTACGTTGGTGATGAAGCT-3’ and 5’-GTCAGTCAAATCTCTACCGG-3’) were used. The real-time PCR was initiated at 95 °C for 10 min, followed by 40 cycles of denaturation for 5 sec at 95 °C, annealing for 30 sec at 60 °C, and elongation for 60 sec at 72 °C. Fluorescence signals were collected at 72 °C during the elongation step. Each DNA template was performed in triplicate. The results were analyzed using the LightCycle480 SW 1.5.1.

### FACS analysis of DNA replication

One-milliliter samples from meiotic cultures were pelleted and resuspended in 70% ethanol. Samples were stored at −20 °C until FACS analysis. Before FACS analysis, cells were washed with 50 mM sodium citrate and resuspended in 0.5 ml of 50 mM sodium citrate containing 0.1 mg/ml RNase A at 30 °C for 2 hrs. Alternatively, 0.5 ml of 50 mM sodium citrate containing 2 μM Sytox Green (for final concentration 1 μM) was added. Samples were briefly sonicated and analyzed on a Becton-Dickinson FACScan analyzer.

## Additional Information

**How to cite this article**: Wang, Q. *et al.* Yeast model identifies *ENTPD6* as a potential non-obstructive azoospermia pathogenic gene. *Sci. Rep.* **5**, 11762; doi: 10.1038/srep11762 (2015).

## Supplementary Material

Supplementary Information
