# Supplementary material for: Yeast model identifies ENTPD6 as a potential non-obstructive azoospermia pathogenic gene
Source: Sci Rep. 2015 Jul 8;5:11762. doi: 10.1038/srep11762 (PMC4495445; doi:10.1038/srep11762)
Supplement: Supplementary Information [file srep11762-s1.doc]

**Supporting Information**

**Yeast model identifies *ENTPD6* as a potential non-obstructive azoospermia** **pathogenic gene**

Qian Wang^1,4†^, Chao Liu^1,4†^, Chaoming Tang^1,4^, Huiping Guo[[1]](#footnote-2), Yujiao Liu^1,5^, Lina Wang^1,4^, Haichao Zhao^1,4^ ,Yongliang Shang^1,4^, Yang Wen^2^, Yuan Lin^2^, Tao Zhou^2^, Zuomin Zhou^2,3^, Wen Dong^5^, Zhibin Hu^2^, Xuejiang Guo^2,3^*****, Jiahao Sha^2,3^*****, Wei Li^1,4^*****

^1^ State Key Laboratory of Reproductive Biology, Institute of Zoology, Chinese Academy of Sciences, Beijing 100101, China

^2^ State Key Laboratory of Reproductive Medicine, Collaborative Innovation Center of Genetics and Development, Nanjing Medical University, Nanjing 210029, China

^3^ Department of Histology and Embryology, Nanjing Medical University, Nanjing 210029, China

^4^ University of Chinese Academy of Sciences, Beijing 100049, China

^5^ College of Marine Life, Ocean University of China, Qingdao 266003, China

^†^ These authors contributed equally to this work

***** Correspondence should be sent to:

Dr. Wei Li

Institute of Zoology, Chinese Academy of Sciences

1 Beichen West Road, Chaoyang District,

Beijing 100101, P.R. China

Email: **leways@ioz.ac.cn**

Tel: 86-10-64807529

FAX: 86-10-64807529,

Dr. Jiahao Sha, Xuejiang Guo

State Key Laboratory of Reproductive Medicine,

Nanjing Medical University,

Nanjing 210029, China

Email: **shajh@njmu.edu.cn; guo_xuejiang@njmu.edu.cn**

Tel: 86-2586862038/86868440

FAX: 86-25-86862908/86868439

**Supplemental Figure legends**

**
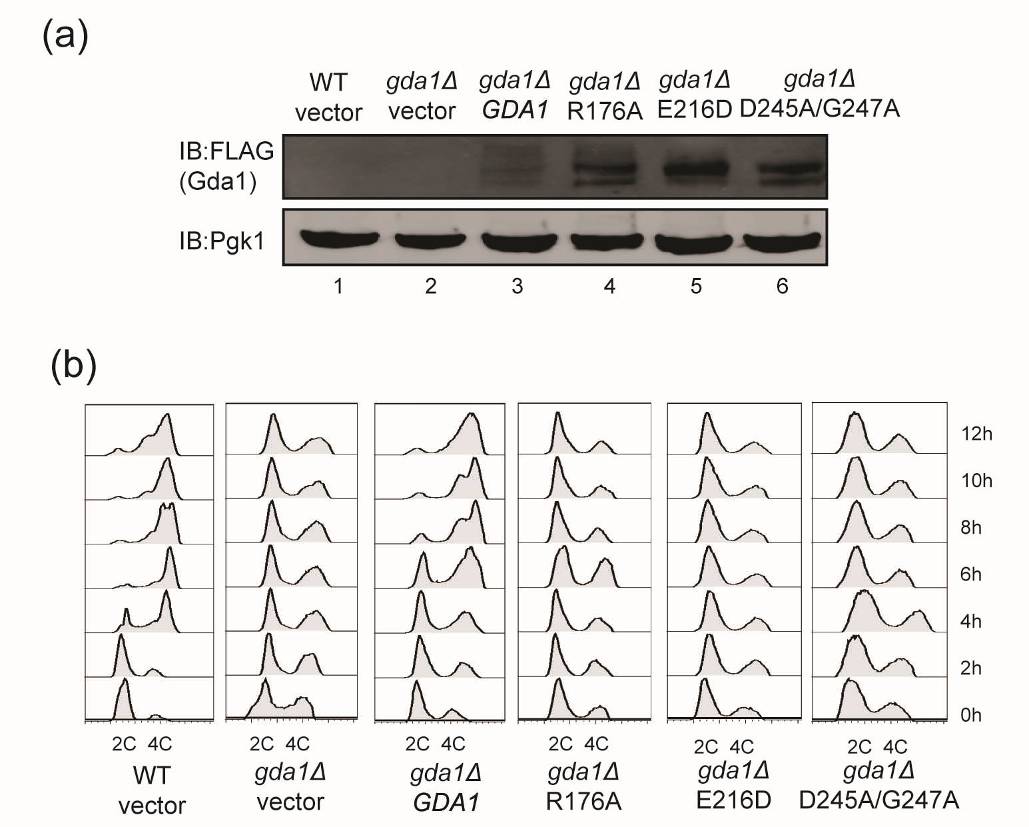
**

**Figure S1. The function of Gda1p in pre-meiotic DNA replication is dependent on its guanosine diphosphatase activity, relate to Figure 1.**

(a) The Gda1p guanosine diphosphatase activity mutant had no effect on their protein expression. WT strain harbored empty vector, gda1*Δ* strains harbored either empty vector or *GDA1*, R176A, E216D, D245A/G247A mutants under the control of their own promoter samples were collected and extracted. The expression of Gda1p was analyzed by immunoblotting with anti-FLAG antibody. Pgk1p served as a loading control.

(b) The disruption of *GDA1* guanosine diphosphatase activity inhibited the pre-meiotic DNA replication during sporulation. WT strain harbored empty vector, gda1*Δ* strains harbored either empty vector or *GDA1*, R176A, E216D, D245A/G247A mutants under the control of their own promoter, were incubated in sporulation medium and samples were collected at different times after sporulation induction. DNA content was analyzed by flow cytometry.

**
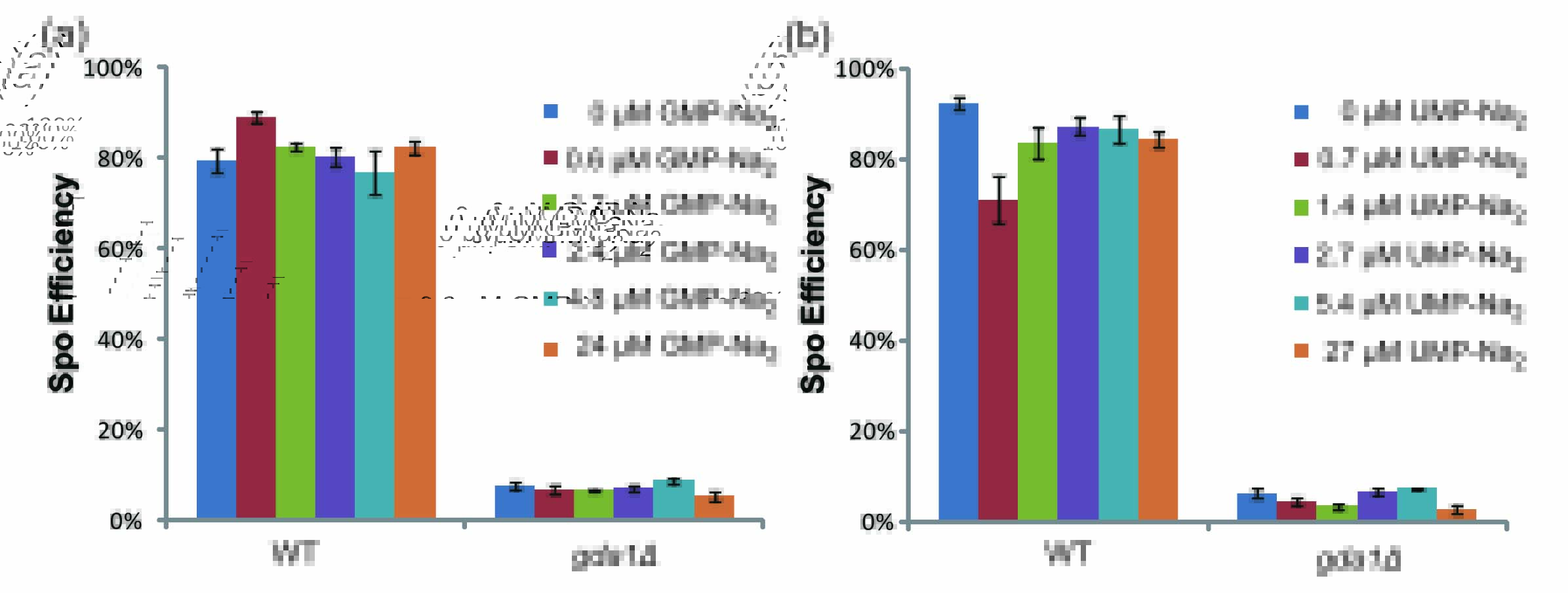
**

**Figure S2** **The additional GMP-Na_2_ and UMP-Na_2_ could not rescue the sporulation defect of the *GDA1* deletion strain.**

(a) The additional GMP-Na_2_ could not rescue the sporulation of the GDA1 deletion strain. WT strain and gda1*Δ* strain were incubated in YPA medium and sporulation medium containing GMP-Na_2_ as indicated concentration. After incubation in sporulation medium for 24h, the sporulation efficiency was counted by staining with DAPI.

(b) The additional UMP-Na_2_ could not rescue the sporulation of the GDA1 deletion strain. WT strain and gda1*Δ* strain were incubated in YPA medium and sporulation medium containing UMP-Na_2_ as indicated concentration. After incubation in sporulation medium for 24h, the sporulation efficiency was counted by staining with DAPI.

**
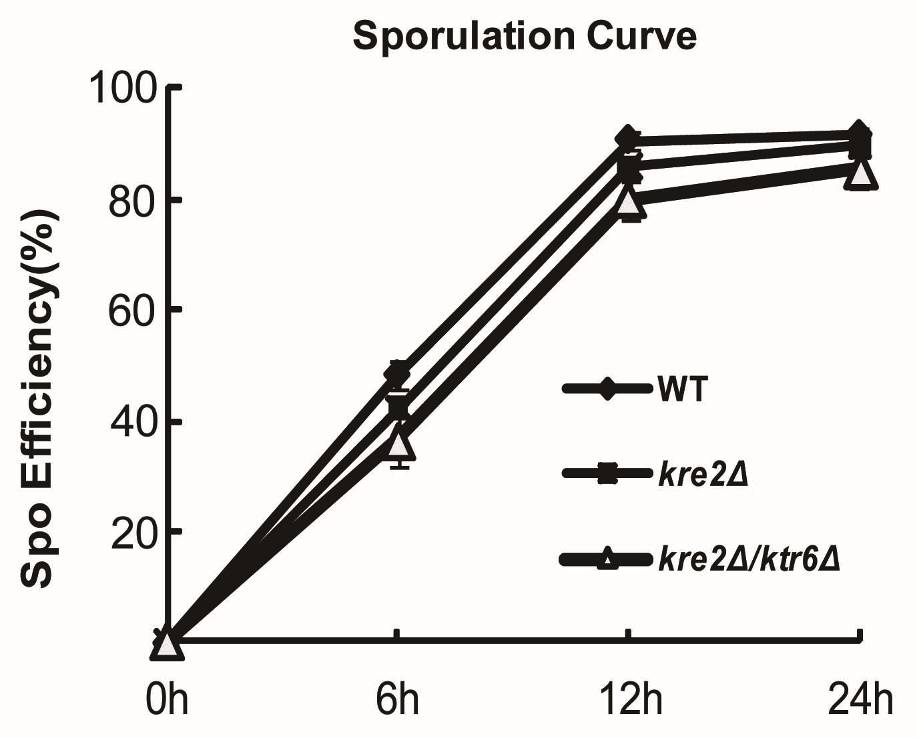
**

**Figure S3** **kre2*Δ* and kre2*Δ*/ktr6*Δ* strain showed no defect in sporulation efficiency compared with the WT strain.**

A sporulation time course to compare the sporulation efficiency of kre2*Δ*, *kre2Δ/ktr6Δ* and WT strains. Diploid yeast cells are deprived of nitrogen, induced to enter sporulation, and stained with DAPI at different times after induction, the dyads and tetrads were counted as sporulated cells.

**
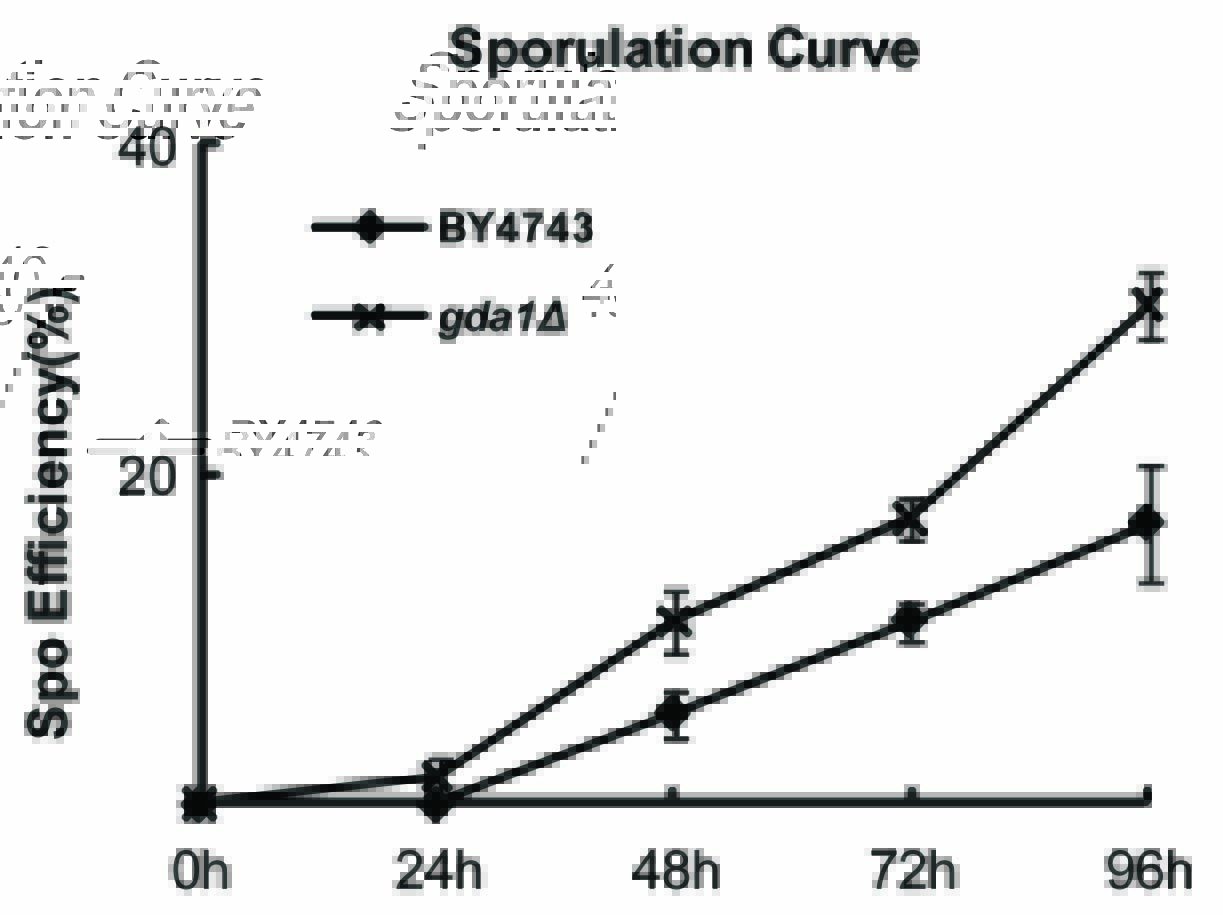
**

**Figure S4** **The sporulation efficiency of gda1*Δ* was not disruptedt in BY4743 background.** A sporulation time course to compare the sporulation efficiency of gda1*Δ* and WT in BY4743 background. Diploid WT and gda1*Δ* BY4743 background yeast cells are deprived of nitrogen, induced to enter sporulation, and stained with DAPI at different times after induction, the dyads and tetrads were counted as sporulated cells.


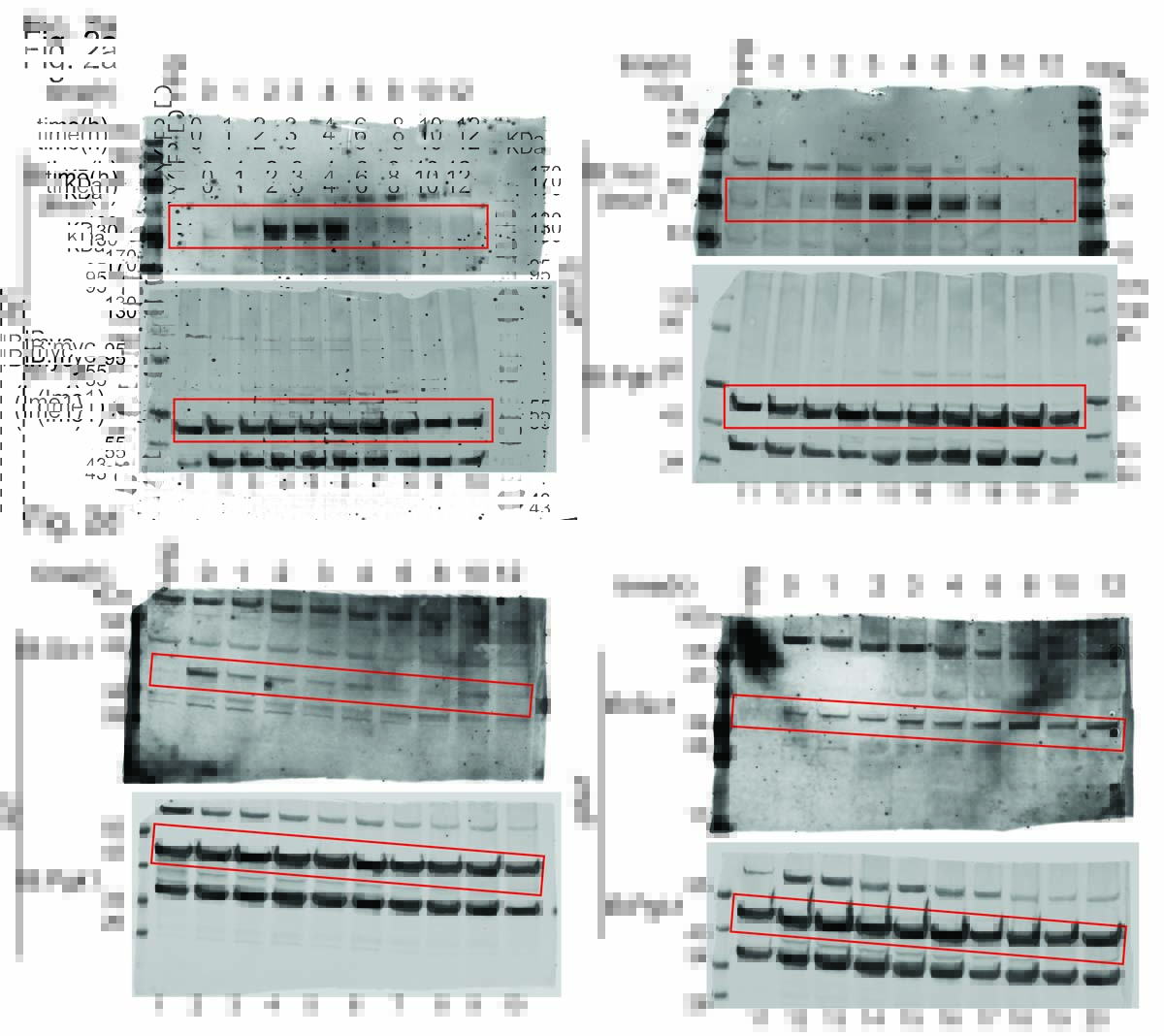


**Figure S5-1** **Full scan of gels related to Figure 2a, 2c.** The samples were loaded onto 8-12% gradient SDS-PAGE gel, and run at 120V for 1.5hrs.


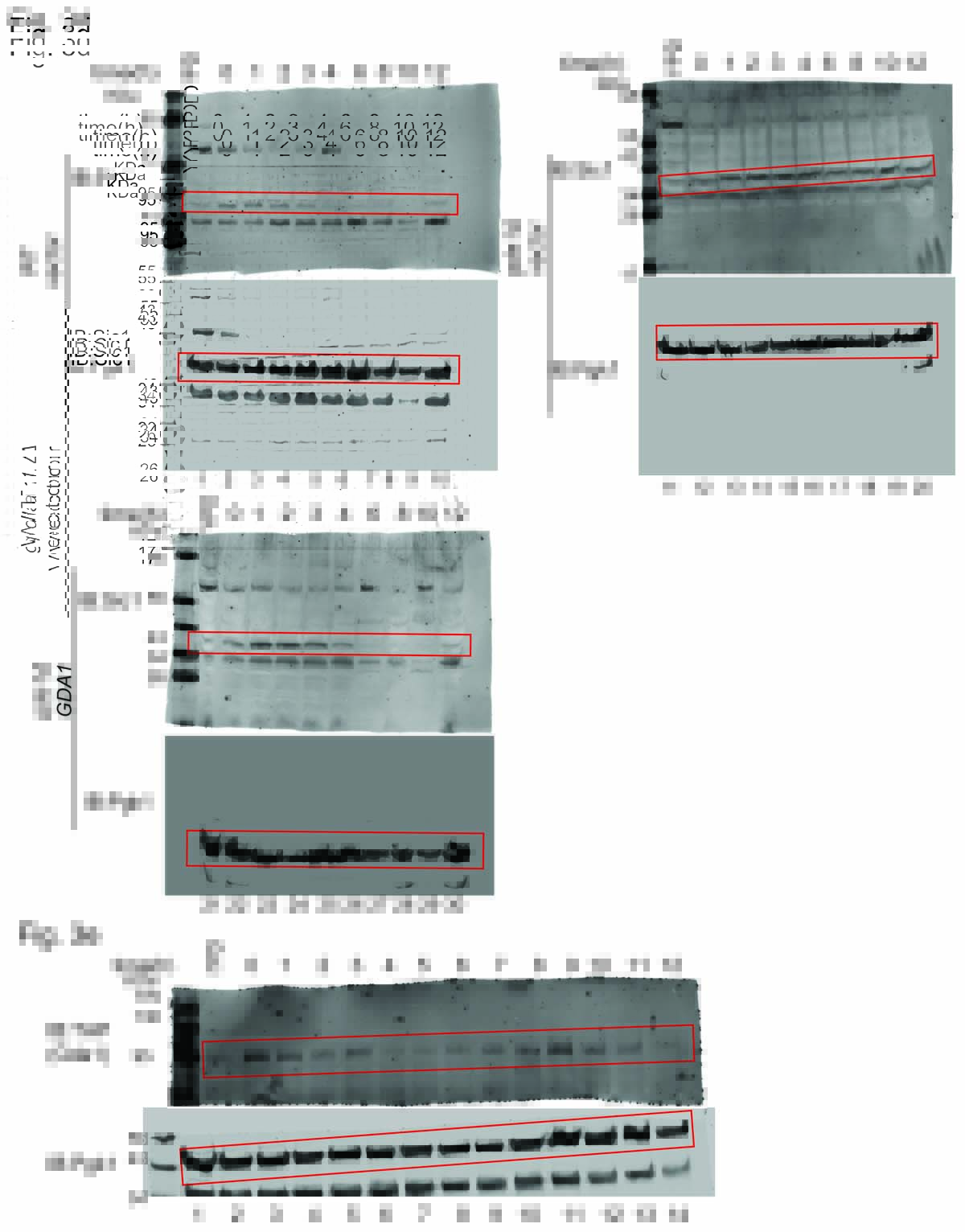


**Figure S5-2** **Full scan of gels related to Figure 3d, 3e.** The samples were loaded onto 8-12% gradient SDS-PAGE gel, and run at 120V for 1.5hrs.


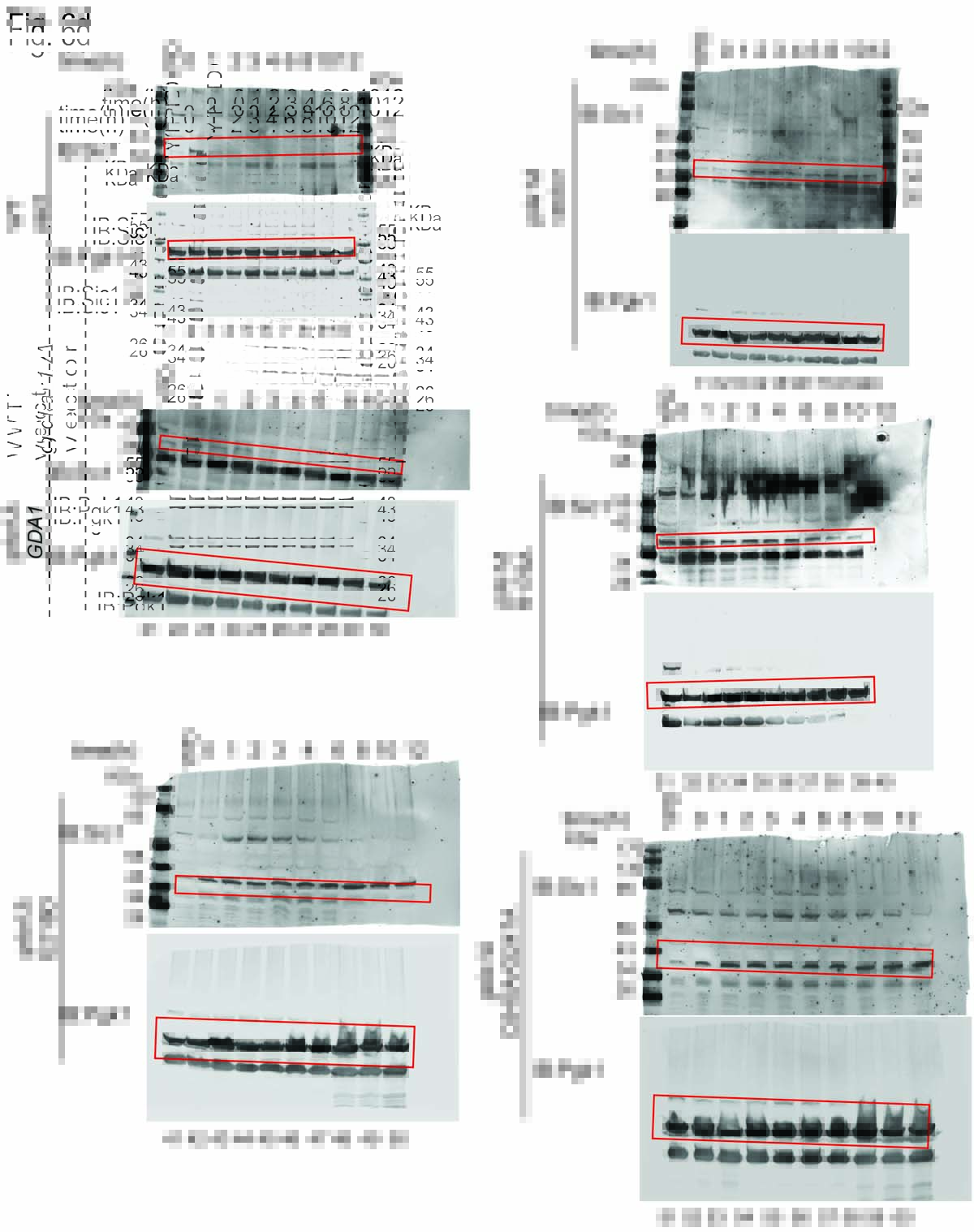


**Figure S5-3** **Full scan of gels related to Figure 6d.** The samples were loaded onto 8-12% gradient SDS-PAGE gel, and run at 120V for 1.5hrs.

**Supplemental Tables**

**
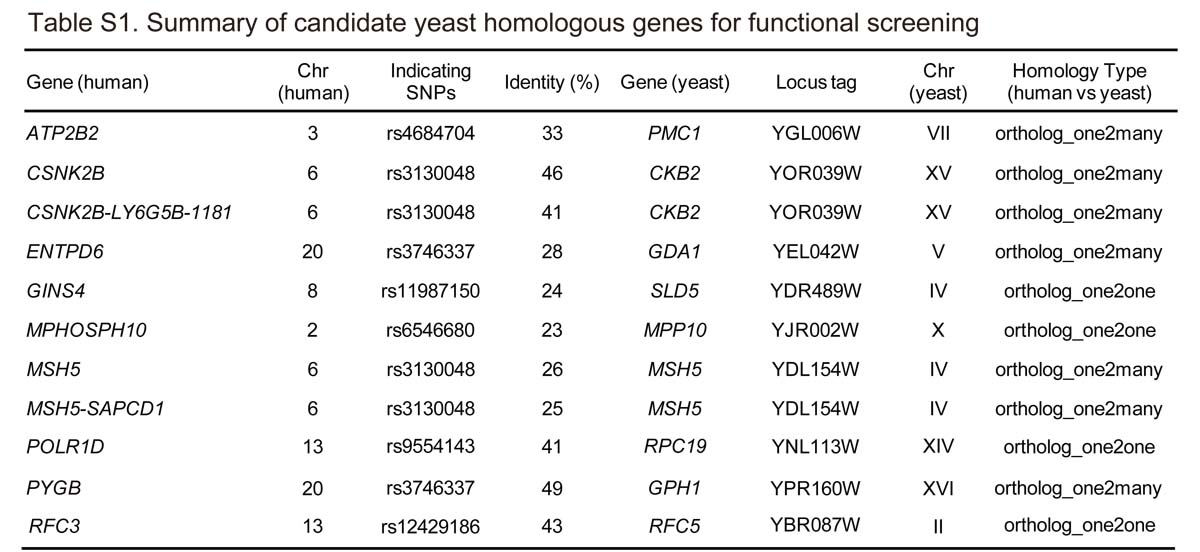
**

**Table S2**. Strains Used in This Study

| Strain Number | Relevant Genotype | Reference |
| --- | --- | --- |
| SK1 | *MATa/*α  *ho::LYS2/ ho::LYS2, lys2/ lys2, ura3/ ura3, leu2::hisG/ leu2::hisG, his3::hisG/ his3::hisG, trp1::hisG/ trp1::hisG,* *ura3::pGPD1-GAL4(848).ER::URA3/ura3::pGPD1-GAL4(848).ER::URA3* | [^1^](#_ENREF_1) |
| LW0066 | *MATa/*α  *ho::LYS2/ ho::LYS2, lys2/ lys2, ura3/ ura3, leu2::hisG/ leu2::hisG, his3::hisG/ his3::hisG, trp1::hisG/ trp1::hisG* | This study |
| BY4743 | *MAT a/α*  *his3Δ1/his3Δ1; leu2Δ0/leu2Δ0; lys2Δ0/LYS2; MET15/met15Δ0; ura3Δ0/ura3Δ0* | [^2^](#_ENREF_2) |
| LW1500 | *MATa/*α  *ho::LYS2/ ho::LYS2, lys2/ lys2, ura3/ ura3, leu2::hisG/ leu2::hisG, his3::hisG/ his3::hisG, trp1::hisG/ trp1::hisG,* *ura3::pGPD1-GAL4(848).ER::URA3/ura3::pGPD1-GAL4(848).ER::URA3,* ***gda1::KanMX4/gda1::KanMX4*** | This study |
| LW1502 | *MATa/*α  *ho::LYS2/ ho::LYS2, lys2/ lys2, ura3/ ura3, leu2::hisG/ leu2::hisG, his3::hisG/ his3::hisG, trp1::hisG/ trp1::hisG,* *ura3::pGPD1-GAL4(848).ER::URA3/ura3::pGPD1-GAL4(848).ER::URA3,* ***pmc1::KanMX4/gda1::KanMX4*** | This study |
| LW1503 | *MATa/*α  *ho::LYS2/ ho::LYS2, lys2/ lys2, ura3/ ura3, leu2::hisG/ leu2::hisG, his3::hisG/ his3::hisG, trp1::hisG/ trp1::hisG,* *ura3::pGPD1-GAL4(848).ER::URA3/ura3::pGPD1-GAL4(848).ER::URA3,* ***ckb2::KanMX4/gda1::KanMX4*** | This study |
| LW1505 | *MATa/*α  *ho::LYS2/ ho::LYS2, lys2/ lys2, ura3/ ura3, leu2::hisG/ leu2::hisG, his3::hisG/ his3::hisG, trp1::hisG/ trp1::hisG,* *ura3::pGPD1-GAL4(848).ER::URA3/ura3::pGPD1-GAL4(848).ER::URA3,* ***gph1::KanMX4/gda1::KanMX4*** | This study |
| LW1507 | *MATa/*α  *ho::LYS2/ ho::LYS2, lys2/ lys2, ura3/ ura3, leu2::hisG/ leu2::hisG, his3::hisG/ his3::hisG, trp1::hisG/ trp1::hisG,* *ura3::pGPD1-GAL4(848).ER::URA3/ura3::pGPD1-GAL4(848).ER::URA3,* ***IME1-3MYC****-TRP1/IME1* | This study |
| LW1508 | *MATa/*α  *ho::LYS2/ ho::LYS2, lys2/ lys2, ura3/ ura3, leu2::hisG/ leu2::hisG, his3::hisG/ his3::hisG, trp1::hisG/ trp1::hisG,* *ura3::pGPD1-GAL4(848).ER::URA3/ura3::pGPD1-GAL4(848).ER::URA3,* ***gda1::KanMX4/gda1::KanMX4****,* ***IME1-3MYC****-TRP1/IME1* | This study |
| LW1509 | *MATa/*α  *ho::LYS2/ ho::LYS2, lys2/ lys2, ura3/ ura3, leu2::hisG/ leu2::hisG, his3::hisG/ his3::hisG, trp1::hisG/ trp1::hisG,* ***kre2::KanMX4/kre2::KanMX4*** | This study |
|  | *MATa/*α  *ho::LYS2/ ho::LYS2, lys2/ lys2, ura3/ ura3, leu2::hisG/ leu2::hisG, his3::hisG/ his3::hisG, trp1::hisG/ trp1::hisG,*  ***kre2::KanMX4/kre2::KanMX4 ktr6::LEU2/ ktr6::LEU2*** | This study |
| LW1510 | *MAT a/α*  *his3Δ1/his3Δ1, leu2Δ0/leu2Δ0, lys2Δ0/LYS2, MET15/met15Δ0, ura3Δ0/ura3Δ0,* ***gda1::KanMX4/gda1::KanMX4*** | This study |
| LW1511 | *MATa/*α  *ho::LYS2/ ho::LYS2, lys2/ lys2, ura3/ ura3, leu2::hisG/ leu2::hisG, his3:: pRS313-HIS3/ his3::hisG, trp1::hisG/ trp1::hisG,* *ura3::pGPD1-GAL4(848).ER::URA3/ura3::pGPD1-GAL4(848).ER::URA3* | This study |
| LW1512 | *MATa/*α  *ho::LYS2/ ho::LYS2, lys2/ lys2,, ura3/ ura3, leu2::hisG/ leu2::hisG, his3::pRS313-HIS3/ his3::hisG, trp1::hisG/ trp1::hisG,* *ura3::pGPD1-GAL4(848).ER::URA3/ura3::pGPD1-GAL4(848).ER::URA3* ***gda1::KanMX4/gda1::KanMX4*** | This study |
| LW1513 | *MATa/*α  *ho::LYS2/ ho::LYS2, lys2/ lys2,, ura3/ ura3, leu2::hisG/ leu2::hisG, his3::* ***pRS313****-HIS3-GDA1-FLAG / his3::hisG, trp1::hisG/ trp1::hisG,* *ura3::pGPD1-GAL4(848).ER::URA3/ura3::pGPD1-GAL4(848).ER::URA3* ***gda1::KanMX4/gda1::KanMX4*** | This study |
| LW1514 | *MATa/*α  *ho::LYS2/ ho::LYS2, lys2/ lys2,, ura3/ ura3, leu2::hisG/ leu2::hisG, his3::* *GDA1-TAP-HIS3/ his3::hisG, trp1::hisG/ trp1::hisG,* *ura3::pGPD1-GAL4(848).ER::URA3/ura3::pGPD1-GAL4(848).ER::URA3* | This study |
| LW1515 | *MATa/*α  *ho::LYS2/ ho::LYS2, lys2/ lys2,, ura3/ ura3, leu2::hisG/ leu2::hisG, his3::* *pRS313-HIS3-GDA1(ΔN1-9)-FLAG/ his3::hisG, trp1::hisG/ trp1::hisG,ura3::pGPD1-GAL4(848).ER::URA3/ura3::pGPD1-GAL4(848).ER::URA3* ***gda1::KanMX4/gda1::KanMX4*** | This study |
| LW1516 | *MATa/*α  *ho::LYS2/ ho::LYS2, lys2/ lys2,, ura3/ ura3, leu2::hisG/ leu2::hisG, his3::* *pRS313-HIS3-GDA1(ΔN10-24)-FLAG / his3::hisG, trp1::hisG/ trp1::hisG,ura3::pGPD1-GAL4(848).ER::URA3/ura3::pGPD1-GAL4(848).ER::URA3* ***gda1::KanMX4/gda1::KanMX4*** | This study |
| LW1517 | *MATa/*α  *ho::LYS2/ ho::LYS2, lys2/ lys2,, ura3/ ura3, leu2::hisG/ leu2::hisG, his3::* *pRS313-HIS3-GDA1(ΔN25-58)-FLAG/ his3::hisG, trp1::hisG/ trp1::hisG,ura3::pGPD1-GAL4(848).ER::URA3/ura3::pGPD1-GAL4(848).ER::URA3* ***gda1::KanMX4/gda1::KanMX4*** | This study |
| LW1518 | *MATa/*α  *ho::LYS2/ ho::LYS2, lys2/ lys2,, ura3/ ura3, leu2::hisG/ leu2::hisG, his3::* *pRS313-HIS3-GDA1(N41D)-FLAG/ his3::hisG, trp1::hisG/ trp1::hisG,ura3::pGPD1-GAL4(848).ER::URA3/ura3::pGPD1-GAL4(848).ER::URA3* ***gda1::KanMX4/gda1::KanMX4*** | This study |
| LW1519 | *MATa/*α  *ho::LYS2/ ho::LYS2, lys2/ lys2,, ura3/ ura3, leu2::hisG/ leu2::hisG, his3::hisG/ his3::hisG, trp1::hisG/ trp1::hisG,* *ura3::pGPD1-GAL4(848).ER::URA3/ura3::pGPD1-GAL4(848).ER::URA3* ***gda1::KanMX4/gda1::KanMX4 pRS313****-HIS3-GDA1(N280D)-FLAG* | This study |
| LW1520 | *MATa/*α  *ho::LYS2/ ho::LYS2, lys2/ lys2,, ura3/ ura3, leu2::hisG/ leu2::hisG, his3::* *pRS313-HIS3-GDA1(N335D)-FLAG/ his3::hisG, trp1::hisG/ trp1::hisG,* *ura3::pGPD1-GAL4(848).ER::URA3/ura3::pGPD1-GAL4(848).ER::URA3* ***gda1::KanMX4/gda1::KanMX4*** | This study |
| LW1521 | *MATa/*α  *ho::LYS2/ ho::LYS2, lys2/ lys2,, ura3/ ura3, leu2::hisG/ leu2::hisG, his3::* *pRS313-HIS3-GDA1(N41D/N280D/N335D) -FLAG/ his3::hisG, trp1::hisG/ trp1::hisG,* *ura3::pGPD1-GAL4(848).ER::URA3/ura3::pGPD1-GAL4(848).ER::URA3* ***gda1::KanMX4/gda1::KanMX4*** | This study |
| LW1522 | *MATa/*α  *ho::LYS2/ ho::LYS2, lys2/ lys2,, ura3/ ura3, leu2::hisG/ leu2::hisG, his3::* *pRS313-HIS3-GDA1(R176A)-FLAG/ his3::hisG, trp1::hisG/ trp1::hisG,ura3::pGPD1-GAL4(848).ER::URA3/ura3::pGPD1-GAL4(848).ER::URA3* ***gda1::KanMX4/gda1::KanMX4*** | This study |
| LW1523 | *MATa/*α  *ho::LYS2/ ho::LYS2, lys2/ lys2,, ura3/ ura3, leu2::hisG/ leu2::hisG, his3::* *pRS313-HIS3-GDA1(E216D)-FLAG/ his3::hisG, trp1::hisG/ trp1::hisG,ura3::pGPD1-GAL4(848).ER::URA3/ura3::pGPD1-GAL4(848).ER::URA3* ***gda1::KanMX4/gda1::KanMX4*** | This study |
| LW1524 | *MATa/*α  *ho::LYS2/ ho::LYS2, lys2/ lys2,, ura3/ ura3, leu2::hisG/ leu2::hisG, his3::* *pRS313-HIS3-GDA1(D245AE247A)-FLAG/ his3::hisG, trp1::hisG/ trp1::hisG,* *ura3::pGPD1-GAL4(848).ER::URA3/ura3::pGPD1-GAL4(848).ER::URA3* ***gda1::KanMX4/gda1::KanMX4*** | This study |

**Table S3. Plasmid**s Used in This Study

| Plasmid | Description | Source |
| --- | --- | --- |
| pRS313 | HIS3 | [^3^](#_ENREF_3) |
| pGDA1 | pRS313-GDA1-FLAG | This study |
| pGDA1(ΔN1-9) | pRS313-GDA1-ΔN1-9-FLAG | This study |
| pGDA1(ΔN10-24) | pRS313-GDA1-ΔN10-24-FLAG | This study |
| pGDA1(ΔN25-58) | pRS313-GDA1-ΔN25-58-FLAG | This study |
| pGDA1(N41D) | pRS313-GDA1-N41D-FLAG | This study |
| pGDA1(N280D) | pRS313-GDA1-N280D-FLAG | This study |
| pGDA1(N335D) | pRS313-GDA1-N335D-FLAG | This study |
| pGDA1(N41D/N280D/N335D) | pRS313-GDA1-N41D/N280D/N335D-FLAG | This study |
| pGDA1(R176A) | pRS313-GDA1-R176A-FLAG | This study |
| pGDA1(E216D) | pRS313-GDA1-E216D-FLAG | This study |
| pGDA1(D245A/G247A) | pRS313-GDA1-D245A/G247A-FLAG | This study |

**Supplemental References**

1. Carlile, T.M. & Amon, A. Meiosis I is established through division-specific translational control of a cyclin. *Cell* **133**, 280-91 (2008).

2. Brachmann, C.B. *et al.* Designer deletion strains derived from Saccharomyces cerevisiae S288C: a useful set of strains and plasmids for PCR-mediated gene disruption and other applications. *Yeast* **14**, 115-32 (1998).

3. Sikorski, R.S. & Hieter, P. A system of shuttle vectors and yeast host strains designed for efficient manipulation of DNA in Saccharomyces cerevisiae. *Genetics* **122**, 19-27 (1989).

1. [↑](#footnote-ref-2)
